# Cost‐Effectiveness and Impact of Depression Treatment Implementation on HIV Outcomes in Western Kenya: A Mathematical Modelling Study

**DOI:** 10.1002/jia2.70186

**Published:** 2026-09-01

**Authors:** Daniel T. Citron, Hae‐Young Kim, Roscoe Kasujja, Khamisi Musanje, Samuel M. Mwalili, Duncan Kioi Gathungu, Peris Wambui, Chikumbi Chambwe, Ingrida Platais, Frey B. Assefa, Mellesia Jeetoo, R. Scott Braithwaite, Anna Bershteyn

**Affiliations:** ^1^ Department of Population Health NYU Grossman School of Medicine New York New York USA; ^2^ School of Psychology Makerere University Kampala Uganda; ^3^ StrongMinds Uganda Kampala Uganda; ^4^ Department of Statistics & Actuarial Science Jomo Kenyatta University of Agriculture and Technology (JKUAT) Nairobi Kenya; ^5^ Center for Health Analysis and Modeling Strathmore University Nairobi Kenya; ^6^ Department of Pure and Applied Mathematics Jomo Kenyatta University of Agriculture and Technology (JKUAT) Nairobi Kenya; ^7^ Division of Mental Health Ministry of Health Nairobi Kenya; ^8^ Centre for Infectious Disease Research in Zambia Lusaka Zambia

**Keywords:** cost‐effectiveness, depression, HIV, interpersonal psychotherapy, mathematical modelling, mental health

## Abstract

**Introduction:**

In eastern, central and southern Africa, HIV is a leading cause of mortality, and both HIV and depression contribute substantially to morbidity. Depression can impede effective HIV treatment and prevention, yet access to depression treatment remains limited. We estimated the impact and cost‐effectiveness of scaling up depression screening and treatment to different population segments in western Kenya .

**Methods:**

We adapted a previously validated HIV transmission model for western Kenya (EMOD‐HIV) to include age‐ and sex‐specific depression incidence, remission and recurrence. The model incorporated depression's effects on HIV risk; HIV testing and linkage to care; antiretroviral therapy (ART) adherence; and ART retention. We evaluated four depression screening and treatment scale‐up strategies for adults aged 15+ years: (1) universal screening/treatment for all adults; (2) co‐administering depression and HIV screening; (3) screening ART recipients; and (4) screening ART recipients with unsuppressed viral load. We assumed 62% depression treatment efficacy and calculated costs from the provider perspective, including US$3.35 for depression screening and US$20.32 per person for psychotherapy; future costs were discounted at 3%, and costs were varied in sensitivity analyses. We calculated incremental cost‐effectiveness ratios as the cost (2024 USD) of depression screening and treatment per overall and HIV‐related disability‐adjusted life‐year (DALY) averted.

**Results:**

Without depression treatment, we projected 129,000 new HIV infections, 95,900 HIV‐related deaths and 1.83 M depression episodes between 2025 and 2035. Universal screening had the highest impact, averting 29.3% (95% CI 29.2%–29.3%) of person‐years lived with depression, 2.8% (95% CI 2.6%–3.1%) of HIV acquisitions and 1.4% (95% CI 1.3%–1.6%) of HIV deaths. The cost‐effectiveness of universal screening and treatment was US$1291 per DALY averted (95% CI: $1288–1296). Screening ART clients was most cost‐effective at $744 per DALY averted (95% CI: $730–760), followed by screening ART clients with unsuppressed viral load at $752 per DALY averted (95% CI: $710–799). Co‐administering HIV and depression was dominated by other strategies.

**Conclusions:**

Integrating depression interventions into HIV care could substantially reduce both depression and HIV burden. Cost‐effective scale‐up could initially focus on individuals with unsuppressed viral load, then to all ART recipients, and finally expand to communities.

## 1 Introduction

Despite considerable progress towards reducing HIV mortality and incidence, HIV remains a major health challenge in eastern, central and southern Africa. At the same time, common mental disorders, particularly depression, are leading contributors to morbidity (i.e. time lived with suboptimal health) in this region [1]. HIV and depression frequently co‐occur, driven by overlapping vulnerabilities such as stigma, substance use, violence and poverty [2, 3]. A recent meta‐analysis estimated that up to 15.3% (95% CI: 12.5%–17.1%) of people living with HIV (PLHIV) in eastern, central and southern Africa experience major depression, which is 2–3 times the rate observed among HIV‐negative individuals [4, 5]. HIV and depression also interact with one another. Symptoms of depression are associated with worse HIV outcomes, including increased risk of HIV acquisition [6]; delayed HIV testing and treatment initiation [7, 8, 9]; lower likelihood of achieving viral load suppression on antiretroviral therapy (ART) [10, 11]; and higher ART dropout rates [12].

Despite the high burden of untreated depression, access to mental health services remains limited, with 85% of affected individuals in the region lacking treatment [13]. Evidence suggests that interpersonal psychotherapy (IPT) is effective when adapted to suit local populations and their cultural contexts in southern and eastern Africa [14, 15]. Studies have also demonstrated the effectiveness of IPT among PLHIV, both for reducing depressive symptoms [16, 17, 18] and for improving ART adherence and viral suppression [19]. Based on this evidence, the World Health Organization recommends IPT as a first‐line treatment for depression in resource‐limited settings [20].

Expanding access to effective depression treatment could improve not only mental health outcomes but also strengthen HIV prevention and care in southern, central and eastern Africa. Kenya has a national mental health policy guiding mental health services, but the Nyanza region in Western Kenya has few specialists and low capacity, and there are no specific policies on service delivery [21, 22, 23, 24, 25]. To better understand the potential impact and cost‐effectiveness of scaling up depression treatment in settings with high HIV prevalence, we adapted a previously validated simulation model of HIV in the Nyanza region of western Kenya—an area with 13.2% HIV prevalence in adults, as estimated in the 2018 KENPHIA survey—to incorporate depression, its interactions with HIV and the potential effects of scaling up depression treatment across different population segments.

We used this model to estimate the potential impact and cost‐effectiveness of integrating depression screening and treatment with HIV screening, HIV care or as a universal service for all adults, for example through community‐based delivery strategies.

## 2 Methods

### 2.1 EMOD‐HIV Simulation Model

We used EMOD‐HIV, a stochastic, individual‐based network transmission model, to simulate HIV outcomes in western Kenya. EMOD‐HIV represents both heterosexual and vertical HIV transmission by generating synthetic populations with realistic demographics. It builds an age‐specific partnership network through which HIV can spread; tracks the progression of the disease in individuals; simulates access and distribution of HIV preventative measures (e.g. traditional and voluntary medical male circumcision) and HIV treatment; and simulates individual treatment response to ART (Figure S1).

The model was previously calibrated to match the HIV prevalence and ART coverage of six high HIV burden counties in western Kenya comprising the former province of Nyanza (Homa Bay, Siaya, Kisumu, Migori, Nyamira and Kisii) [26, 27]. Calibration was performed using stochastic gradient descent to match modelled outputs against region‐specific demographic projections; HIV prevalence; ART distribution; and voluntary male medical circumcision (VMMC) coverage (Figure S2). The final calibrated model included an ensemble of 250 best‐fit trajectories, each with different parameters that account for uncertainty underlying the calibration target data. Additional details on the calibration procedure and chosen parameters are available in Table S1.

### 2.2 EMOD‐HIV‐Depression Simulation Model

We extended EMOD‐HIV to simulate the natural history of depression alongside HIV. Drawing from established clinical frameworks for the course of depression, we augmented each simulated adult with an individual‐level state‐transmission model comprised of four discrete stages: depression‐naïve, depressed, recovered without treatment and recovered following treatment (Figure S1 and Table S2) [28]. Individuals entered the simulation in the depression‐naïve state and could develop depression at an incidence rate determined by age, sex and HIV status. Recovery was modelled with an exponentially distributed duration with a mean of 9 months, based on clinical estimates [28, 29]. After recovery, individuals remained at risk of relapse, allowing for multiple recurrent depression episodes over their lifetime. We did not incorporate direct mortality due to depression (e.g. suicide) due to scarcity of data and depression burden being predominantly attributable to morbidity (i.e. years lived in suboptimal health). We calibrated depression incidence to match age‐ and sex‐specific major depression prevalence estimated for Kenya (Figure S3 and Table S2) [30, 31].

Our EMOD‐HIV‐Depression model incorporated bi‐directional interactions between HIV and depression (Figure S2; further parameter details in Table S3), based on a review of studies conducted in southern and eastern Africa. Depression prevalence among PLHIV is known to be 2–3 times as high as among people living without HIV; accordingly, we adjusted depression incidence among PLHIV to reflect this elevated depression prevalence [32]. Depression also influenced HIV transmission and treatment outcomes: individuals with depression had a 1.67‐fold (95% CI: 1.67–1.68) increased risk of HIV acquisition and transmission, reflecting a combination of bio‐behavioural factors such as less consistent condom use [6]. Individuals with depression were 48.3% (95% CI: 30.1%–66.5%) less likely to receive routine HIV testing, causing delays to ART initiation [9]. From a recent meta‐analysis of the effects of depression on ART outcomes, we assumed that depressed individuals who used ART had a 1.20‐fold (95% CI: 0.83–1.72) increased risk of viral load non‐suppression [33]. Additionally, individuals on ART with a history of depression (recovered with or without treatment) had a slightly increased risk of viral load non‐suppression, 1.19 (95% CI: 1.11–1.26) [11]. Lastly, depression was associated with higher rates of ART discontinuation, with depressed individuals interrupting ART at a rate 2.4 (95% CI 1.27–4.5) times higher than individuals without depression [12]. Further model parameter details may be found in Table S3.

### 2.3 Description of Depression Screening and Treatment

Our model included depression screening and treatment for different population segments. For individuals eligible to receive depression screening, we selected screening dates randomly throughout the year for each eligible individual. We assumed 92% sensitivity and 90% specificity of screening, based on PHQ‐9 diagnostic performance in Uganda [34]. Individuals with depression who screened positive were offered treatment modelled on response to IPT. We assumed a delay between positive screening and treatment of less than 1 month (one simulation time step). We assumed 62% treatment efficacy [35], whereby successful treatment reduced the mean duration of a depressive episode from 9 to 3 months, in accordance with clinical estimates [35, 36]. Treatment status also reduced depression relapse rates: Individuals who recovered without treatment experienced relapse at the same rate as the age‐, sex‐ and HIV status‐specific incidence, while those who recovered following treatment had lower depression relapse risk, with an odds ratio of 0.5 (95% CI 0.12−2.02) [37]. We assumed this effect lasted until the start of the next depression episode. Depression treatment did not directly affect modelled HIV outcomes; rather, its effects on HIV outcomes emerged through reductions in depression and the associated interactions between depression and HIV.

### 2.4 Depression Screening and Treatment Strategy Scenarios

We simulated four different strategies for expanding access to depression screening and treatment among adults (aged 15+). In the universal screening scenario, all adults were screened, representing the widest possible scale‐up of depression services, for example screening all adults in their communities. Second, in the HIV‐depression co‐screening strategy, depression screening occurred during routine HIV testing. Third, in the ART client screening strategy, all PLHIV on ART received depression screening. Fourth, in the Viral Load Monitoring strategy, only PLHIV on ART with unsuppressed viral load were screened for depression. Lastly, we modelled a scenario with no screening or treatment as a baseline for comparison. For each strategy, screening‐eligible individuals would receive depression screening once per year. We simulated each strategy from the start of the HIV pandemic in 1985 until 2035, with depression screening and treatment introduced in 2025. All evaluations were conducted over a 10‐year time horizon from 2025 through 2035.

We assumed pre‐2025 levels of HIV testing, ART coverage and HIV prevention service coverage were maintained through 2035. Results reflect the means of 250 simulated best‐fit trajectories, with 95% confidence intervals estimated using 500 bootstrap samples obtained from the rsample package in R.

## 3 Evaluations

### 3.1 Metrics of Disease Burden

For each scenario, we estimated the number of new HIV acquisitions, HIV‐related deaths and person‐years lived with depression, as well as the total disability‐adjusted life‐years (DALYs) associated with HIV and depression from 2025 to 2035. DALYs were calculated using disability weights of 0.274 for HIV, 0.078 for HIV while on ART and 0.396 for major depression, with a 3% annual discount rate applied from 2025 onwards [38, 39]. We applied a single HIV disability weight rather than stage‐specific weights as a parsimonious modelling simplification. Due to the paucity of reliable data, we did not include additional mortality due to depression outside of its effect on HIV, for example increased rates of suicide.

### 3.2 Economic Evaluation

Using an ingredient‐based costing analysis from the provider perspective, we estimated the costs of depression screening (US$3.35 per screening) and treatment (US$20.32 per course), accounting for health worker time spent administering assessments and delivering eight interpersonal therapy sessions (Table S4). Additionally, we assumed an all‐inclusive cost of US$319 per person‐year on ART [40] in order to take into account potential healthcare cost savings from averted HIV infections. For each screening and treatment strategy, we estimated the total HIV acquisitions, HIV‐related deaths, person‐years lived with HIV while on ART, and DALYs related to HIV and depression from 2025 to 2035. Using these cost estimates, we calculated the incremental cost‐effectiveness ratios (ICERs), representing the cost per DALY and the cost per HIV‐related DALY averted through depression screening and treatment. We compared our cost‐effectiveness estimates with published marginal cost‐effectiveness thresholds for Kenya: US$698 per DALY averted from Woods et al. [41] and US$832 per DALY averted from Ochalek et al. [42] (all costs reported in 2025 US dollars).

### 3.3 Sensitivity Analysis

To assess the robustness of our results and main contributors to uncertainty, we conducted a univariate sensitivity analysis to evaluate the how assumptions related to key model parameters affected outcomes—depression screening sensitivity and specificity; depression treatment efficacy; increased HIV risk among people living with depression; increased depression incidence among PLHIV; reduced ART uptake, adherence and retention for PLHIV living with depression; and costs of depression screening and treatment. Values were varied across their uncertainty intervals from reported trials or meta‐analyses (Table S3). We varied the assumed costs of testing and treatment by ±20%, and varied the discounting rate from 0% to 6%.

We also modelled a scenario reflecting post‐2025 changes to HIV services following the loss of US foreign assistance for Kenya's HIV programme. Using methods described in [43], we assumed a 41% reduction in ART coverage, HIV teesting, and VMMC beginning in 2025 to asses how these service disruptions could affect our cost‐effectiveness methods.

We generated a cost‐effectiveness acceptability curve (CEAC) to assess overall uncertainty in the comparative cost‐effectiveness of each strategy [44]. We generated CEACs to characterize decision uncertainty across a range of cost‐effectiveness thresholds. CEACs show, for each threshold value, the probability that each strategy is the most cost‐effective. The threshold values reported from the CEAC are the crossover points at which the strategy with the highest probability of being cost‐effective changes.

### 3.4 Ethical Considerations

This study employed mathematical and simulation modelling and did not involve human participants, clinical trials or the use of protected personal information. All analyses were conducted using publicly available aggregated data.

## 4 Results

### 4.1 Impact of Depression Treatment

Of 4.89 million (95% CI 4.89–4.90 million) individuals aged 15 and older represented in the model at the start of 2025, a total of 556,000 (95% CI 550,000–562,000) were PLHIV and 358,000 (95% CI 357,000–358,000) were people living with depression. A total of 74,300 (95% CI 73,700–75,100) were living with both depression and HIV. In the scenario without depression screening or treatment, between the years 2025 and 2035, we estimated 129,000 (95% CI 128,000–132,000) cumulative HIV acquisitions, 95,900 (95% CI 94,800–96,900) HIV‐related deaths and 1.833 million (95% CI 1.832–1.834 million) person‐years lived with depression.

**FIGURE 1:**
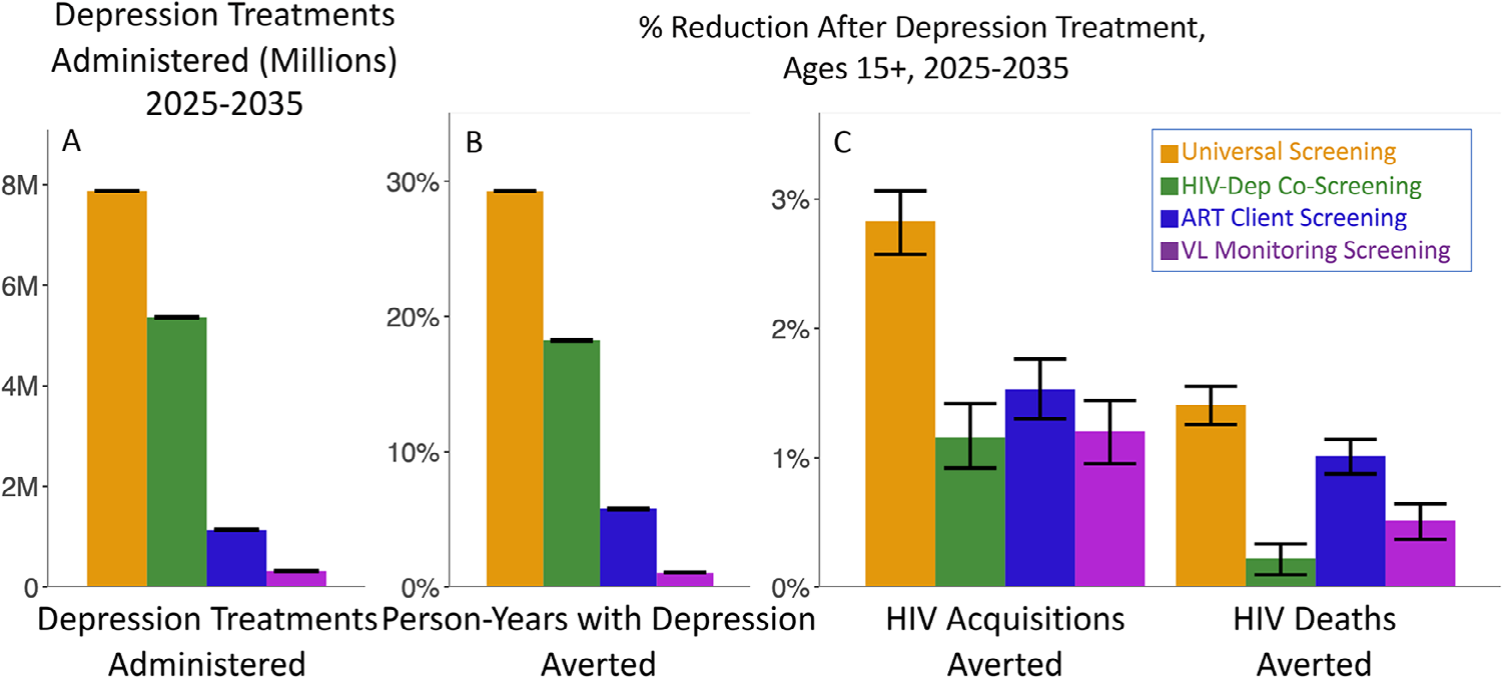
Impact of depression treatment on depression and HIV. (A) Number of depression treatments administered for each screening strategy. (B) Percent reduction in person‐years lived with depression due to depression treatment. (C) Percent reduction in HIV acquisitions and HIV‐related deaths due to depression treatment.

The universal screening strategy, which targeted the largest population for depression care, required 48.7 million (95% CI 48.7−48.8 million) screenings and 7.88 million (95% CI 7.87−7.88 million) treatments over the simulation period (Table 1 and Table S5). This strategy yielded the greatest impact, averting 529,900 (95% CI 529,400–530,400) person‐years lived with depression (29.3% reduction, 95% CI 29.2%–29.3%), 1350 (95% CI 1220–1480) HIV acquisitions (2.8% reduction, 95% CI 2.6%–3.1%) and 3680 (95% CI 3370–3990) HIV‐related deaths (1.4% reduction, 95% CI 1.3%–1.6%) (Figure 1 and Table S5). For comparison, the viral load monitoring screening strategy, which targeted the smallest population, required 1.09 million (95% CI 1.07–1.10 million) screenings and 318,000 (95% CI 315,000–321,000) treatments. It averted 20,300 (95% CI 19,810–20,800) person‐years lived with depression (1.1% reduction, 95% CI 1.0%–1.1%), 498 (95% CI 357–636) HIV acquisitions (1.2% reduction, 95% CI 1.0%–1.4%) and 1570 (95% CI 1300–1880) HIV‐related deaths (0.5% reduction, 95% CI 0.4%–0.6%) (Figure 1, Table 1 and Table S5).

**TABLE 1:** Simulation outcomes.

Metric	Baseline	Universal screening	HIV‐depression co‐screening	ART client screening	VL monitoring screening
Depression screenings (millions)	0 (95% CI 0–0)	48.7 (95% CI 48.7–48.78)	34.7 (95% CI 34.6–34.8)	5.14 (95% CI 5.09–5.19)	1.09 (95% CI 1.07–1.10)
Depression treatments (millions)	0	7.88 (7.87–7.88)	5.36 (5.35–5.37)	1.14 (1.13–1.15)	0.318 (0.315–0.321)
Person‐years of depression (millions)	1.83 (1.83–1.83)	1.30 (1.30–1.30)	1.50 (1.50–1.51)	1.73 (1.73–1.73)	1.81 (1.81–1.81)
Person‐years of depression averted	0	530,000 (529,400–530,400)	330,000 (329,000–331,000)	105,000 (104,00–106,000)	2,0300 (19,800–20,800)
HIV acquisitions	129,000 (128,000–132,000)	126,000 (124,000–128,000)	128,000 (126,000–130,000)	127,000 (126,000–130,000)	128,000 (126,000–130,000)
HIV acquisitions averted	0	1350 (1220–1480)	221 (107–354)	970 (865–1090)	498 (357–636)
HIV deaths	95,900 (94,800–96,900)	94,500 (93,400–95,600)	95,600 (94,600–96,600)	94,900 (94,000–95,900)	95,400 (94,400–96,300)
HIV deaths averted	0	3680 (3370–3990)	1560 (1190–1870)	2010 (1690–2330)	1570 (1300–1880)
HIV‐related DALYs	978,000 (967,000–989,000)	971,000 (961,000–980,000)	976,000 (967,000–986,000)	973,000 (962,000–984,000)	975,000 (965,000–984,000)
HIV‐related DALYs averted	0	6880 (6230–7570)	1920 (1250–2530)	4270 (3690–4870)	2550 (1960–3150)
Total DALYs (millions)	1.70 (1.69–1.71)	1.49 (1.48–1.50)	1.57 (1.56–1.58)	1.66 (1.65–1.67)	1.69 (1.68–1.70)
Total DALYs averted	0	217,000 (216,000–217,000)	133,000 (132,000–133,000)	45,800 (45,000–46,700)	10,600 (10,000–11,200)
Cost of screening, $millions USD	0	142 (142–142)	101 (100–101)	15.01 (14.84–15.16)	3.17 (3.14–3.20)
Cost of treatment, $millions USD	0	139 (139–139)	94.3 (94.2–94.5)	20.1 (19.9–20.3)	5.63 (5.57–5.69)
Cost of ART, $millions USD	1424 (1409–1440)	1426 (1410–1441)	1424 (1410–1437)	1424 (1410–1440)	1424 (1409–1440)
ICER	NA	1291 (1296–1288)	1467 (1474–1460)	744 (760–730)	752 (799–710)

### 4.2 Economic Evaluation

**FIGURE 2:**
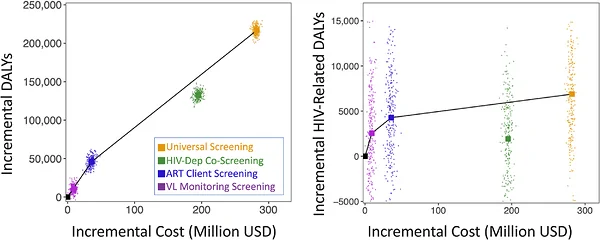
Incremental cost‐effectiveness curves. Incremental DALYs (left) and HIV‐related DALYs (right) averted per incremental cost of depression screening and treatment and the cost of ART. Solid boxes show the mean estimates, and circles show results from each of 250 individual simulations.

Based on average ICERs, the most cost‐effective strategy was ART client screening, with an ICER of US$744 per DALY averted (95% CI US$730–760). Viral load monitoring screening had a similar ICER (US$752; 95% CI US$710–799) and lay close to the efficiency frontier (Figure 2, left panel). The next strategy on the efficiency frontier was universal screening (ICER: US$1291; 95% CI US$1288–1296). HIV‐depression co‐screening was dominated. When considering only the HIV‐related health burden averted through depression screening (Figure 2, right panel), the most cost‐effective strategy was viral load monitoring screening, with an ICER of US$3530 per HIV‐related DALY averted (95% CI 2850–4700). ART client screening followed (8270; 95% CI US$7270–9520), then universal screening (US$41,000; 95% CI 37,700–45,300).

**FIGURE 3:**
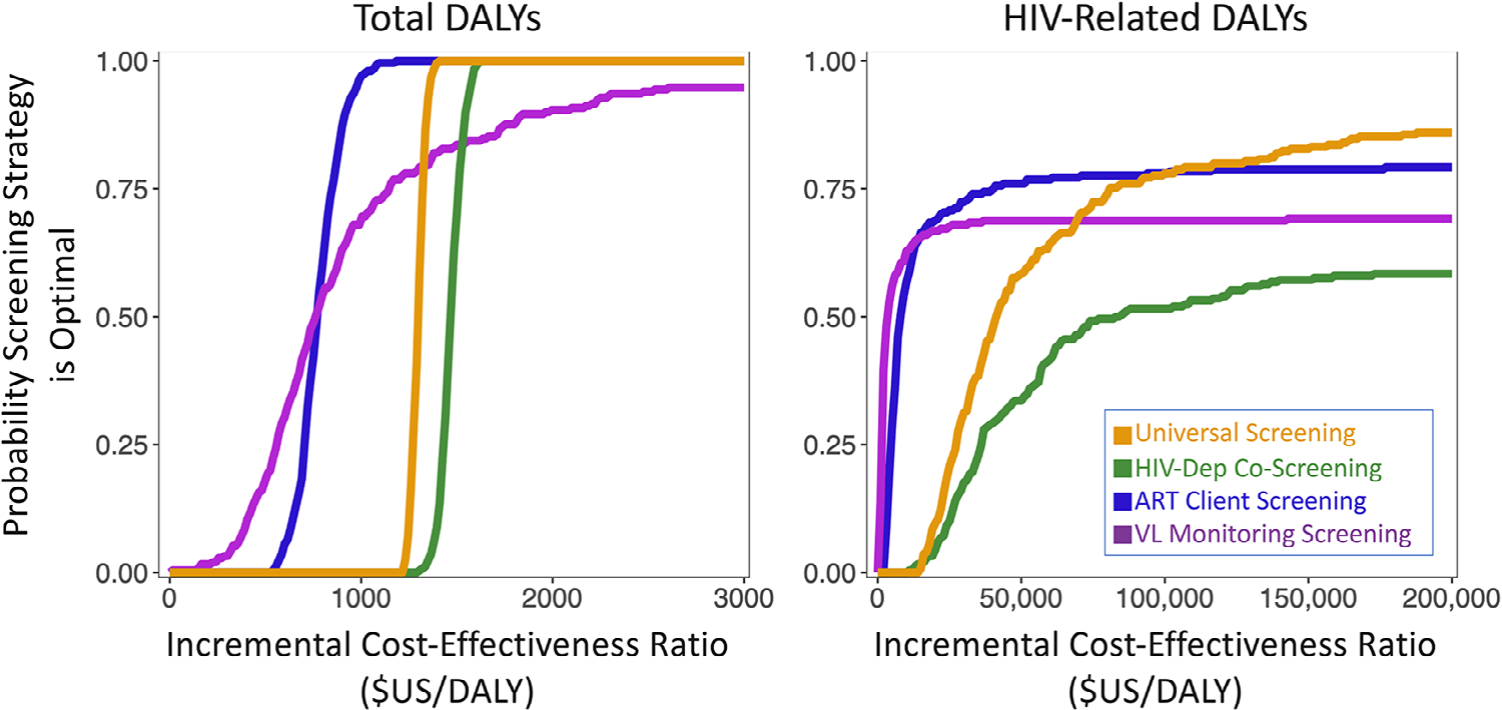
Cost‐effectiveness acceptability curves. Cost‐effectiveness acceptability curves (CEACs) corresponding to the efficiency frontiers in Figure 2, showing the probability that each screening strategy is optimally efficient for a given incremental cost‐effectiveness ratio (ICER). The left panel shows the CEAC for the total DALYs averted, and the right panel shows the CEAC for HIV‐related DALYs only.

CEACs (Figure 3) were used to examine decision uncertainty across a range of willingness‐to‐pay thresholds. In these curves, the reported threshold values represent crossover points at which the strategy with the highest probability of being cost‐effective changes. For DALYs averted, viral load monitoring screening had the highest probability of being cost‐effective at thresholds below US$775 per DALY averted; ART client screening had the highest probability between US$775 and US$1396 per DALY averted; and universal screening had the highest probability above US$1396 per DALY averted. For HIV‐related DALYs averted, the corresponding crossover points were US$15,000 and US$103,500.

### 4.3 Sensitivity Analysis

**FIGURE 4:**
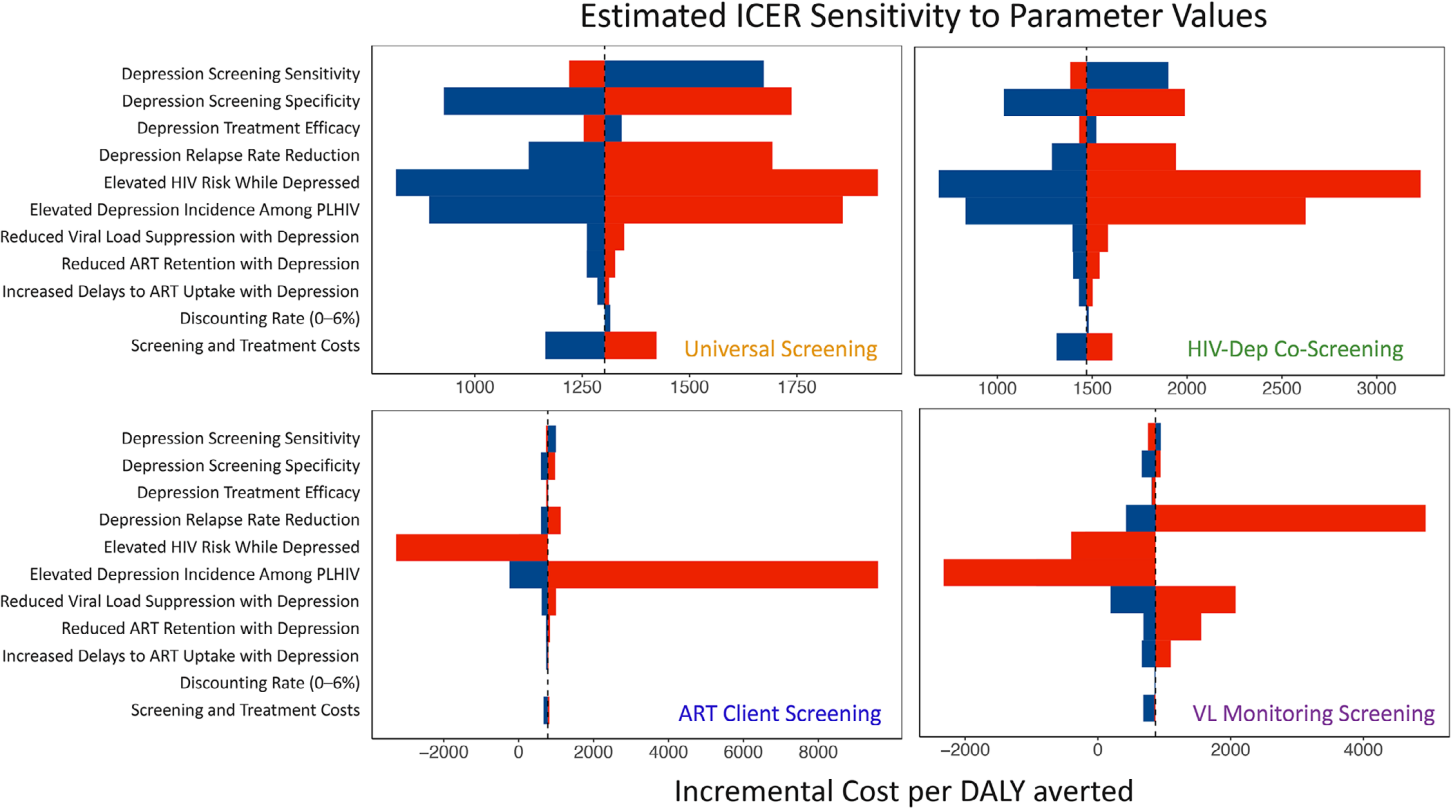
Sensitivity analysis. Univariate sensitivity analysis for the universal screening scenario. Dashed vertical lines represent the baseline ICER estimates, and coloured bars represent how ICER estimates vary under different assumptions. We varied each parameter according to its confidence intervals (Table S3). Blue bars show the upper bound of the parameter value's confidence interval, and red bars show the lower bound.

In the univariate sensitivity analysis (Figure 4), we varied depression screening sensitivity from 0.67 to 1.0. As a result, the estimated universal screening ICER ranged from US$1674 to US$1221, while estimated ART client screening ICER ranged from US$977 to US$719 (Table S6). We varied the depression screening specificity from 0.78 to 1.0. As a result, the estimated universal screening ICER ranged from US$1738 to US$930, and the estimated ART client screening ICER ranged from US$968 to US$605, respectively. We varied the depression treatment efficacy from 56% to 68% and found the estimated universal screening ICER ranged from US$1341 to US$1250, and the ART client screening ICER ranged from US$816 to US$731. There was similarly little change in the results when varying the effect of depression on HIV testing and ART outcomes. Two model parameters—the effect of depression on viral load non‐suppression and the effect of treatment on depression relapse—had 95% confidence intervals that did not exclude no effect. In sensitivity analyses, varying these parameters across their confidence intervals did not materially change the results. Two bi‐directional interactions between depression and HIV—increased depression incidence among PLHIV and increased HIV risk among people with depression—did result in substantial variation in estimated cost‐effectiveness but did not reorder the efficiency frontier.

Our results were robust to assumptions around discounting and depression screening and treatment costs. Varying the discount rate from 0% to 6% yielded ICERs of US$1280–1315 for universal screening, US$1456–1481 for HIV‐depression co‐screening, US$731–792 for ART client screening and US$764–794 for viral load monitoring screening. When depression treatment costs were varied by ±20%, the corresponding ICERs ranged from US$1043 to 1561, US$1182 to 1770, US$643 to 949 and US$700 to 1031, respectively. The ordering of the efficiency frontier did not change for either of these sensitivity analyses.

Lastly, in the scenario reflecting the 41% reduction in HIV services as a consequence of the loss of foreign aid, we found higher ICER estimates for each scenario: US$1301 (95% CI US$1294–1308) for universal screening, US$1448 (95% CI US$1426–1470) for HIV‐depression co‐screening, US$832 (95% CI US$726–961) for viral load monitoring screening and US$797 (95% CI US$768–829) for ART client screening. We interpret these increases as reflecting a reduced overall impact of depression treatment in scenarios with elevated HIV incidence, prevalence and mortality due to a loss of HIV prevention and treatment services.

## 5 Discussion

We used a simulation model to assess the impact of screening and treating depression in western Kenya, a region with high HIV prevalence. The model incorporated the bi‐directional interactions between HIV and depression, allowing us to estimate the health impacts of depression screening and treatment on both HIV and depression. We found that the most cost‐effective strategy was to integrate depression care for all PLHIV on ART. This strategy was slightly more cost‐effective than targeting PLHIV not currently achieving viral load suppression, although the difference was not statistically significant. Optimal expansion of depression screening would first target all virally unsuppressed PLHIV on ART, then expand to all PLHIV on ART, then to universal screening.

Interpreting the cost‐effectiveness of depression care depends on opportunity costs—the benefit forgone from funding other health interventions instead. Two earlier studies have estimated the marginal cost‐effectiveness threshold for health interventions in Kenya, which serve as useful benchmarks. In 2025 US dollars, Woods et al. estimated a threshold of US$698 per DALY averted [41], and Ochalek et al. estimated US$832 per DALY averted [42]. In our study, the ICER for depression treatment among PLHIV on ART with unsuppressed viral load was US$752 (95% CI US$710–799) per DALY averted—between the two benchmarks. This suggests depression treatment may be a cost‐effective option for consideration in Kenyan general healthcare, and highlights the need for further research to reduce uncertainty around both threshold estimates and intervention costs. HIV‐specific ICERs (i.e. ICERs only considering health benefits associated with HIV outcomes) were high, suggesting that if funds are earmarked for the specific goal of reducing HIV burden, providing depression care for PLHIV is unlikely to be a cost‐effective way to advance this goal, when compared to many HIV treatment and prevention options in need of funding.

Scaling depression screening and treatment requires not only funding but also trained mental health providers. While our analysis focused on cost‐effectiveness relative to a funding constraint, it is also important to compare strategies that make the best use of available human resources. For instance, StrongMinds, a Uganda‐based non‐governmental organization, has scaled depression care by training former clients as providers of IPT operating in rural community settings. Their programme offers context‐specific mental health support without requiring clinicians or existing health system infrastructure. The programme has reduced the cost of depression care to US$23 per client [45], comparable to estimates in our analysis, suggesting that a similar programme may prove to be cost‐effective when implemented in western Kenya if its effectiveness can be maintained at scale.

Our methodology has several limitations. Depression prevalence and incidence data for eastern, central and southern Africa are limited: To calibrate our depression model, we searched the Global Health Data Exchange, white and grey literature, and consulted with mental health leadership in Kenya, but only identified one population‐wide survey on depression conducted in Kenya from 2004. As our sensitivity analysis showed, estimates of depression incidence, directly informed by this single survey, are critical to estimating the impact of depression treatment. Our model must be revised and refined as new data on the prevalence and incidence of depression in the region become available. We also did not model years of life lost due to depression, which may lead to an underestimation of total DALYs averted. Although the model was applied to Kenya, some PHQ‐9 and treatment‐effect parameters were drawn from Ugandan studies because Kenya‐specific data were limited. Future analyses should update these parameters as local data become available. We assumed that depression screening and treatment capacity was available from 2025 onwards and did not explicitly model a gradual implementation phase. In addition, our cost estimates reflect service delivery costs only and do not include broader system‐level investments needed for scale‐up, including training, supervision and infrastructure. This likely underestimates total costs, especially for universal screening and other strategies requiring broad early expansion. While a phased implementation model would likely increase early costs and reduce short‐term benefits, we do not expect it to alter the overall conclusion that more targeted strategies, particularly ART client screening, are more favourable initial approaches than universal scale‐up. Finally, we modelled only one treatment modality—IPT—as a first‐line intervention. Future analyses could incorporate a broader range of treatment options, including alternative therapies (e.g. cognitive behavioural therapy) and pharmacotherapy options.

## 6 Conclusions

Depression and HIV will continue to overlap and exacerbate one another. Expanding access to mental health support in western Kenya could reduce untreated depression and simultaneously improve HIV outcomes. Our findings suggest that integrating mental health screening and treatment into existing HIV care infrastructure—particularly as part of counselling and follow‐up for those struggling to maintain viral load suppression—may be a cost‐effective first step towards scaling depression care for all those in need.

## Author Contributions

**Roscoe Kasujja**: conceptualization, writing – original draft, writing – review and editing, methodology, investigation. **Khamisi Musanje**: conceptualization, methodology, writing – original draft, writing – review and editing. **Duncan Kioi Gathungu**: conceptualization, methodology, writing – original draft, writing – review and editing. **Anna Bershteyn**: conceptualization, methodology, investigation, software, writing – review and editing, writing – original draft, funding acquisition, supervision. **Frey B. Assefa**: conceptualization, methodology, investigation, writing – original draft, writing – review and editing, formal analysis, data curation. **Peris Wambui**: conceptualization, methodology, writing – original draft, writing – review and editing. **Chikumbi Chambwe**: conceptualization, methodology, writing – original draft, writing – review and editing. **Hae‐Young Kim**: writing – original draft, writing – review and editing, conceptualization, methodology, data curation. **Ingrida Platais**: conceptualization, methodology, project administration, writing – original draft, writing – review and editing. **Mellesia Jeetoo**: conceptualization, methodology, formal analysis, investigation, writing – original draft, writing – review and editing, data curation. **Daniel T. Citron**: methodology, investigation, validation, formal analysis, conceptualization, writing – original draft, writing – review and editing, software, project administration, data curation. **R. Scott Braithwaite**: methodology, conceptualization, writing – original draft, writing – review and editing. **Samuel M. Mwalili**: conceptualization, methodology, writing – original draft, writing – review and editing.

## Funding

Research reported in this publication was supported by the National Institute of Mental Health of the National Institutes of Health under award numbers R01MH124478 and RF1MH130238.

## Disclaimer

The content is solely the responsibility of the authors and does not necessarily represent the official views of the National Institutes of Health.

## Conflicts of Interest

AB discloses grants from the US National Institutes of Health, New York City Department of Health and Mental Hygiene, Foundation for Innovative New Diagnostics, and Gates Foundation, and personal consulting for Gates Ventures, outside of the reported work. All other authors have nothing to disclose.

## Supporting information




**Supporting file 1**: jia270186‐supp‐info‐0001‐SuppMat.docx

**Supporting file 2**: jia270186‐supp‐info‐0002‐SuppMat.xlsx

**Supporting file 3**: jia270186‐supp‐info‐0003‐SuppMat.xlsx

**Supporting file 4**: jia270186‐supp‐info‐0004‐SuppMat.xlsx

## Data Availability

The data that support the findings of this study are available in the Supplementary Material of this article.
