# Supplementary material for: Cost‐Effectiveness and Impact of Depression Treatment Implementation on HIV Outcomes in Western Kenya: A Mathematical Modelling Study
Source: J Int AIDS Soc. 2026 Sep 1;29(9):e70186. doi: 10.1002/jia2.70186 (PMC13535130; doi:10.1002/jia2.70186)
Supplement: Supplementary file 1 [file JIA2-29-e70186-s001.docx]

# Supplementary Material: Cost-effectiveness and impact of depression treatment implementation on HIV outcomes in western Kenya: a mathematical modeling study

## Supplementary Figure 1

Compartmental representation of HIV-Depression model, with interactions between HIV and depression. EMOD-HIV itself is an agent-based model, but the compartments shown in the top row represent the natural history of HIV, linkage to treatment, and treatment response. The compartments shown in the bottom row represent that natural history of depression.

## Supplementary Figure 2

EMOD-HIV Model Calibration to HIV prevalence by age and sex in Kenya. Calibration target data are taken from DHS surveys conducted in 2003, 2007, 2008, and 2012, and the Kenya PHIA survey conducted in 2018.

## Supplementary Figure 3

Depression model calibration to estimated depression prevalence by age and sex in Kenya. Calibration targets are from the Kenya World Health Survey conducted in 2004.

## Supplementary Figure 4

Time series plots comparing the simulation trajectories of six metrics: number of depression screenings performed, number of depression treatments administered, annual person-years lived with depression, persons living with HIV (PLHIV), annual HIV acquisitions, and annual HIV-related deaths.

## Supplementary Figure 5

Time series plots comparing the simulation trajectories of six metrics for the additional set of scenarios reflecting the post-2025 changes to HIV services in Kenya following the loss of US-based foreign assistance supporting Kenya’s HIV program: number of depression screenings performed, number of depression treatments administered, annual person-years lived with depression, persons living with HIV (PLHIV), annual HIV acquisitions, and annual HIV-related deaths. Comparing this figure to the baseline shown in Supplementary Figure 4, we note an increase in HIV acquisitions, deaths, and PLHIV due to the 41% reduction in HIV services – ART, testing, and VMMC.

## Supplementary Table 1: Simulation model design features

| **Simulation Model** | **Description** | **Citations** |
| --- | --- | --- |
| EMOD-HIV: an agent-based network  transmission of HIV | Calibrated to age and sex-specific HIV prevalence and ART distribution data in western Kenya and validated to predict community-level HIV incidence in western Kenya | Model structure: Bershteyn et al. (2012)^S1^  Calibration: Bershteyn et al. (2018)^S2^  Validation: Jewell et al. (2022)^S3^ |
| Compartmental model of depression: Natural history of depression | Individuals began in a depression-naive state and could experience episodes of depression with an incidence rate dependent on their age, sex, and HIV status. Recovered individuals were susceptible to relapse or recurrence, allowing for multiple recurrent episodes of depression throughout their lifetime. | Compartmental model structure:  Richards (2011)^S4^  Duration of depression episodes:  Ten Have (2017)^S5^  Ferrari et al. (2013)^S6^  Recurrence of depression:  Beshai et al. (2011)^S7^  Bockting et al. (2015)^S8^ |
| Compartmental model of depression: Prevalence calibration | Calibrated to age and sex-specific depression data. Assume that the rate of relapse equals incidence rates | World Health Organization (WHO). Kenya World Health Survey 2004. Geneva, Switzerland: World Health Organization (WHO), (2005)^S9^ |

## Supplementary Table 2

Depression prevalence estimates are taken from the Kenya World Health Survey 2004.^S9^ Annual depression incidence is derived by fitting the state transition model illustrated in Figure 1 to depression prevalence data and are reported below in terms of per person-year.

| **Year** | **Gender** | **Age** | **Depression Prevalence**  **(95% CI)** | **Annual Depression Incidence,  HIV Positive  (95% CI)** | **Annual Depression Incidence,  HIV Negative  (95% CI)** |
| --- | --- | --- | --- | --- | --- |
| 2004 | Male | [18:25) | 0·03  (0·0–0·69) | 0·0867  (0·0–0·202) | 0·043  (0·0–0·100) |
| 2004 | Male | [25:30) | 0·069  (0·0–0·14) | 0·189  (0·0–0·391) | 0·093  (0·0–0·191) |
| 2004 | Male | [30:40) | 0·098  (0·0–0·20) | 0·248  (0·0–0·531) | 0·121  (0·0–0·257) |
| 2004 | Male | [40:45) | 0·087  (0·013–0·16) | 0·215  (0·037–0·416) | 0·105  (0·018–0·200) |
| 2004 | Male | [45:50 | 0·11  (0·045–0·18) | 0·299  (0·130–0·509) | 0·146  (0·064–0·246) |
| 2004 | Male | [50:60) | 0·13  (0·034–0·22) | 0·370  (0·090–0·648) | 0·179  (0·045–0·315) |
| 2004 | Male | [60:99) | 0·2  (0·13–0·27) | 0·595  (0·382–0·796) | 0·287  (0·185–0·394) |
| 2004 | Female | [18:25) | 0·065  (0·024–0·11) | 0·179  (0·065–0·309) | 0·088  (0·033–0·152) |
| 2004 | Female | [25:30) | 0·086  (0·041–0·13) | 0·213  (0·103–0·329) | 0·104  (0·051–0·160) |
| 2004 | Female | [30:40) | 0·067  (0·026–0·11) | 0·157  (0·060–0·274) | 0·077  (0·030–0·133) |
| 2004 | Female | [40:45) | 0·12  (0·058–0·17) | 0·332  (0·164–0·488) | 0·162  (0·081–0·238) |
| 2004 | Female | [45:50 | 0·13  (0·037–0·22) | 0·363  (0·092–0·650) | 0·176  (0·046–0·317) |
| 2004 | Female | [50:60) | 0·079  (0·03–0·12) | 0·212  (0·081–0·328) | 0·103  (0·040–0·158) |
| 2004 | Female | [60:99) | 0·051  (0·0043–0·099) | 0·141  (0·010–0·283) | 0·069  (0·005–0·137) |

## Supplementary Table 3: Model input assumptions related to interactions between HIV and depression, and effects of treatment

| **Feature Name** | **Description** | **Parameter value** | **Parameter value source** | **Supporting sources** |
| --- | --- | --- | --- | --- |
| Mean duration of depressive episode | Assume exponentially distributed depressive episode duration | 9 months (95% CI 1- 21 months) | Ten Have et al. (2017)^S5^ | Ferrari et al. (2013)^S6^ |
| Relative risk of depression among PLHIV compared to HIV-negative cohort | Depression incidence is also calibrated such that prevalence of depression is higher among PLHIV.  Other supporting sources discuss stigma, substance use, and other HIV-related causes of depression | 2 (95% CI 1-3) | Lofgren et al. (2019)^S10^ | Ciesla and Roberts, (2001)^S11^,Earnshaw et al. (2014),  Gamarel et al. (2017),  Earnshaw et al. (2018),  Stockton et al. (2020)^S12–S15^ |
| Elevated HIV acquisition and transmission risk among people experiencing depression | Cited source is a longitudinal study measuring relative risk of HIV acquisition among women ages 13-21. Assume relative risk is similar across age groups for all genders for HIV acquisition and transmission.  Other supporting sources discuss reduced preventative measures such as condom use and PrEP among depressed individuals. | 1·675 (95% CI [1·674, 1·676]) relative risk of HIV acquisition and transmission | Goin et al. (2021)^S6^ | Meade and Sikkema (2005),  Velloza et al. (2018), Velloza et al. (2020),  Velloza et al. (2021)^S17–S20^ |
| Increased delays to ART initiation for among people experiencing depression |  | 2·2 (95% CI [1·01-4·8]) odds of delayed testing. | Rane et al. (2018)^S21^ | Truong et al. (2021),  Ramirez-Avila et al. (2012), Turan et al. (2014)^S22–S24^ |
| Decreased rate of adherence to ART for individuals experiencing depression, increased risk of viral load non-suppression | Calculated based on odds ratio of achieving viral load suppression (VLS) based on depression status | 81·6% (95% CI: 80·5-82·8%) of non-depressed individuals achieve VLS 77·9% (95% CI: 70·5%-83·8%) of depressed individuals achieve VLS | Uthman et al. (2014)^S25^ |  |
| Decreased rate of adherence to ART for individuals with lifetime history of depression (not currently experiencing depression) |  | 19% increased probability of failing to achieve VLS | Haas et al. (2023) ^S26^ | Nakimuli-Mpungu et al. (2009),  Hoffmann et al. (2016),  Blain et al. (2020),  Stockton et al. (2021),  Regan et al. (2021),  Rosenthal et al. (2021)^S27–S31^ |
| Increased rates of dropout from ART usage among people experiencing depression | Relative rate of ART dropout for people experiencing depression | 2·4 (95% CI: 1·27-4·52) | Krumme et al. (2015)^S32^ |  |
| Depression Screening Sensitivity | Sensitivity at a PHQ-9 cutoff of 10 used as mean for simulation purposes· Sensitivity at a PHQ-9 cutoff of 5 used as lower bound for sensitivity analysis; perfect sensitivity used as upper bound for sensitivity analysis | 0·92 (0·67-1·0) | Kaggwa et al. (2022)^S33^ | Akena et al. (2013)^S34^ |
| Depression Screening Specificity | Specificity at a PHQ-9 cutoff of 10 used as mean for simulation purposes· Specificity at a PHQ-9 cutoff of 5 used as lower bound for sensitivity analysis; perfect Specificity used as upper bound for sensitivity analysis | 0·89 (0·78-1·0) | Kaggwa et al. (2022)^S33^ | Akena et al. (2013)^S34^ |
| Depression Treatment Efficacy |  | 0·62 (95% CI 0·56-0·68) | Cuijpers et al. (2014)^S35^ |  |
| Reduction in Duration of Depressive Episode |  | 0•41 (95% CI 0•38-0•43) | Cuijpers et al. (2021)^S36^ |  |
| Reduction in Relapse Rate post-Treatment | Odds ratio of interpersonal therapy relative to placebo after 12 months | 0·5 (95% CI 0·12-2·02) | Zhou et al. (2023)^S37^ | Vittengl and Jarrett (2015)^S38^ |

## Supplementary Table 4 – Costing Analysis

## Supplementary Table 5 – Summary of Simulation Results

## Supplementary Bibliography

S1	Bershteyn A, Klein DJ, Wenger E, Eckhoff PA. Description of the EMOD-HIV Model v0. 7. *arXiv preprint arXiv:12063720* 2012.

S2	Bershteyn A, Mutai KK, Akullian AN, Klein DJ, Jewell BL, Mwalili SM. The influence of mobility among high-risk populations on HIV transmission in Western Kenya. *Infect Dis Model* 2018; **3**: 97–106.

S3	Jewell BL, Balzer LB, Clark TD, *et al.* Predicting HIV Incidence in the SEARCH Trial: A Mathematical Modeling Study. *JAIDS Journal of Acquired Immune Deficiency Syndromes* 2021; **87**: 1024–31.

S4	Richards D. Prevalence and clinical course of depression: A review. *Clinical Psychology Review* 2011; **31**: 1117–25.

S5	ten Have M, Penninx BWJH, Tuithof M, *et al.* Duration of major and minor depressive episodes and associated risk indicators in a psychiatric epidemiological cohort study of the general population. *Acta Psychiatrica Scandinavica* 2017; **136**: 300–12.

S6	Ferrari AJ, Charlson FJ, Norman RE, *et al.* The Epidemiological Modelling of Major Depressive Disorder: Application for the Global Burden of Disease Study 2010. *PLoS ONE* 2013; **8**. DOI:10.1371/journal.pone.0069637.

S7	Beshai S, Dobson KS, Bockting CLH, Quigley L. Relapse and recurrence prevention in depression: Current research and future prospects. *Clinical Psychology Review* 2011; **31**: 1349–60.

S8	Bockting CL, Hollon SD, Jarrett RB, Kuyken W, Dobson K. A lifetime approach to major depressive disorder: The contributions of psychological interventions in preventing relapse and recurrence. *Clinical Psychology Review* 2015; **41**: 16–26.

S9	World Health Organization (WHO). Kenya World Health Survey 2004. 2005.

S10	Lofgren SM, Bond DJ, Nakasujja N, Boulware DR. Burden of Depression in Outpatient HIV-Infected adults in Sub-Saharan Africa; Systematic Review and Meta-analysis. *AIDS Behav* 2019; published online Nov 13. DOI:10.1007/s10461-019-02706-2.

S11	Ciesla JA, Roberts JE. Meta-Analysis of the Relationship Between HIV Infection and Risk for Depressive Disorders. *AJP* 2001; **158**: 725–30.

S12	Earnshaw VA, Smith LR, Shuper PA, Fisher WA, Cornman DH, Fisher JD. HIV stigma and unprotected sex among PLWH in KwaZulu-Natal, South Africa: A longitudinal exploration of mediating mechanisms. *AIDS Care - Psychological and Socio-Medical Aspects of AIDS/HIV* 2014; **26**: 1506–13.

S13	Gamarel KE, Kuo C, Boyes ME, Cluver LD. The dyadic effects of HIV stigma on the mental health of children and their parents in South Africa. *J HIV AIDS Soc Serv* 2017; **16**: 351–66.

S14	Earnshaw VA, Kidman RC, Violari A. Stigma, Depression, and Substance Use Problems Among Perinatally HIV-Infected Youth in South Africa. *AIDS Behav* 2018; **22**: 3892–6.

S15	Stockton MA, Pence BW, Mbote D, *et al.* Associations among experienced and internalized stigma, social support, and depression among male and female sex workers in Kenya. *Int J Public Health* 2020; **65**: 791–9.

S16	Goin DE, Pearson RM, Craske MG, *et al.* Depression and incident HIV in adolescent girls and young women in HIV prevention trials network 068: Targets for prevention and mediating factors. *American Journal of Epidemiology* 2021; **189**: 422–32.

S17	Meade CS, Sikkema KJ. HIV risk behavior among adults with severe mental illness: a systematic review. *Clin Psychol Rev* 2005; **25**: 433–57.

S18	Velloza J, Baeten JM, Haberer J, *et al.* Effect of Depression on Adherence to Oral PrEP Among Men and Women in East Africa. *J Acquir Immune Defic Syndr* 2018; **79**: 330–8.

S19	Velloza J, Heffron R, Amico KR, *et al.* The Effect of Depression on Adherence to HIV Pre-exposure Prophylaxis Among High-Risk South African Women in HPTN 067/ADAPT. *AIDS and Behavior* 2020; **24**: 2178–87.

S20	Velloza J, Hosek S, Donnell D, *et al.* Assessing longitudinal patterns of depressive symptoms and the influence of symptom trajectories on PrEP persistence among adolescent girls in HPTN 082. *Journal of the International AIDS Society* 2021; **24**: 70.

S21	Rane MS, Hong T, Govere S, *et al.* Depression and anxiety as risk factors for delayed care-seeking behavior in human immunodeficiency virus-infected individuals in South Africa. *Clinical Infectious Diseases* 2018; **67**: 1411–8.

S22	Truong M, Rane M, Vander Stoep A, Drain PK. Depression and anxiety as a barrier to art initiation in kwazulunatal, South Africa. *Topics in Antiviral Medicine* 2019; **27**: 357s.

S23	Ramirez-Avila L, Regan S, Giddy J, *et al.* Depressive symptoms and their impact on health-seeking behaviors in newly-diagnosed HIV-infected patients in Durban, South Africa. *AIDS and Behavior* 2012; **16**: 2226–35.

S24	Turan B, Stringer KL, Onono M, *et al.* Linkage to HIV care, postpartum depression, and HIV-related stigma in newly diagnosed pregnant women living with HIV in Kenya: a longitudinal observational study. *BMC Pregnancy Childbirth* 2014; **14**. DOI:10.1186/s12884-014-0400-4.

S25	Uthman OA, Popoola TA, Yahaya I, Uthman MMB, Aremu O. The cost-utility analysis of adult male circumcision for prevention of heterosexual acquisition of HIV in men in sub-Saharan Africa: a probabilistic decision model. *Value in Health* 2011; **14**: 70–9.

S26	Haas AD, Lienhard R, Didden C, *et al.* Mental Health, ART Adherence, and Viral Suppression Among Adolescents and Adults Living with HIV in South Africa: A Cohort Study. *AIDS and Behavior* 2023. DOI:10.1007/s10461-022-03916-x.

S27 Nakimuli-Mpungu E, Mutamba B, Othengo M, Musisi S. Psychological distress and adherence to highly active anti-retroviral therapy (HAART) in Uganda: a pilot study. *Afr Health Sci* 2009; **9 Suppl 1**: S2-7.

S28	Hoffmann CJ, Cohn S, Mashabela F, *et al.* Treatment Failure, Drug Resistance, and CD4 T-Cell Count Decline among Postpartum Women on Antiretroviral Therapy in South Africa. *Journal of Acquired Immune Deficiency Syndromes* 2016; **71**: 31–7.

S29	Blain M, Richardson BA, Kinuthia J, *et al.* Psychosocial Factors, Condomless Sex, and Detectable Viral Load in HIV-Positive Women in Serodiscordant Couples in Nairobi, Kenya. *AIDS and Behavior* 2020; **24**: 3346–58.

S30	Regan M, Muhihi A, Nagu T, *et al.* Depression and Viral Suppression Among Adults Living with HIV in Tanzania. *AIDS and Behavior* 2021; **25**: 3097–105.

S31 Rosenthal MA, Wanje G, Richardson BA, *et al.* A Prospective Study of Depressive Symptoms, Condomless Sex, and HIV Viral Load in HIV-Positive Female Sex Workers in Kenya. *AIDS and Behavior* 2021; **25**: 3047–56.

S32	Krumme AA, Kaigamba F, Binagwaho A, Murray MB, Rich ML, Franke MF. Depression, adherence and attrition from care in HIV-infected adults receiving antiretroviral therapy. *Journal of Epidemiology and Community Health* 2015; **69**: 284–9.

S33	Kaggwa MM, Najjuka SM, Ashaba S, Mamun MA. Psychometrics of the Patient Health Questionnaire (PHQ-9) in Uganda: A Systematic Review. *Front Psychiatry* 2022; **13**: 781095.

S34	Akena D, Joska ,John, Obuku ,Ekwaro A., and Stein DJ. Sensitivity and specificity of clinician administered screening instruments in detecting depression among HIV-positive individuals in Uganda. *AIDS Care* 2013; **25**: 1245–52.

S35	Cuijpers P, Karyotaki E, Weitz E, Andersson G, Hollon SD, van Straten A. The effects of psychotherapies for major depression in adults on remission, recovery and improvement: A meta-analysis. *Journal of Affective Disorders* 2014; **159**: 118–26.

S36	Cuijpers P, Karyotaki E, Ciharova M, Miguel C, Noma H, Furukawa TA. The effects of psychotherapies for depression on response, remission, reliable change, and deterioration: A meta-analysis. *Acta Psychiatrica Scandinavica* 2021; **144**: 288–99.

S37	Zhou Y, Zhao D, Zhu X, *et al.* Psychological interventions for the prevention of depression relapse: systematic review and network meta-analysis. *Transl Psychiatry* 2023; **13**: 1–9.

S38	Vittengl JR, Clark LA, Dunn TW, Jarrett RB. Reducing Relapse and Recurrence in Unipolar Depression: A Comparative Meta-Analysis of Cognitive–Behavioral Therapy’s Effects. *J Consult Clin Psychol* 2007; **75**: 475–88.
